# The Degree of Acceptance of the Disease by Patients After a Diagnosis of Lung Cancer and Their Hope

**DOI:** 10.3390/jcm14124356

**Published:** 2025-06-18

**Authors:** Agnieszka Waleczko, Bożena Baczewska, Beata Barańska, Maria Mielnik-Błaszczak, Krzysztof Leśniewski

**Affiliations:** 1 Department of Internal Medicine and Internal Medicine in Nursing, Faculty of Health Sciences, Medical University of Lublin, Chodźki 7, 20-093 Lublin, Poland; waleczkoagnieszka@gmail.com; 2 Institute of Health, The State University of Applied Sciences in Przemyśl, ul. Książąt Lubomirskich 6, 37-700 Przemyśl, Poland; bbaranska1@o2.pl; 3 Department of Paediatric Dentistry, Medical University of Lublin, Chodźki 6, 20-093 Lublin, Poland; mielnikmb@gmail.com; 4 Department of Orthodox Theology, Faculty of Theology, The John Paul II Catholic University of Lublin, Al. Racławickie 14, 20-950 Lublin, Poland; lesni@kul.pl

**Keywords:** attitudes and acceptance of the disease, diagnosis, lung cancer, hope

## Abstract

**Background/Objectives**: The aim of this study was twofold: first, to assess the extent to which patients diagnosed with lung cancer accept their condition and, second, to characterize the profiles and dimensions of the hope experienced by these patients. **Methods**: In order to achieve the aforementioned goals, the following research tools were utilized: the NCN-36 scale to examine hope, the AIS to ascertain the acceptance of one’s condition, and the KI scale to present socio-demographic–temperamental variables. **Results**: The patients exhibited an average level of acceptance of their disease while simultaneously demonstrating a high level of hope. Cluster analysis identified four groups of patients that differed significantly in terms of disease acceptance and felt hope (*p* < 0.001). Significantly higher acceptance of the disease was found in urban patients (*p* = 0.038) and those with higher education (*p* = 0.011), while lower acceptance was noted in those aged over 75 (*p* = 0.006). In turn, the experienced hope was influenced by variables such as age, place of residence, education, housing situation (living alone or with family), overall pace of activity, basic mood, and social and professional status. **Conclusions:** The researchers identified four distinct attitudes among the patients based on their disease-related experiences. These attitudes were categorized as follows: non-accepting–fearful, indifferent–desperate, non-accepting–fighting, and accepting–trusting.

## 1. Introduction

Malignant cancer constitutes one of the most significant health and social challenges facing contemporary populations. Of all cancer types, lung cancer is associated with the highest mortality rate. Moreover, the majority of lung cancer patients are diagnosed at an advanced stage and typically undergo complex treatment regimens [1]. This often leads to heightened psychological symptoms, including distress, fear of recurrence, and uncertainty about the future, all of which can have a profound negative impact on patients’ overall well-being and quality of life [2,3]. The diagnosis and treatment of lung cancer frequently disrupt multiple dimensions of a patient’s daily life, undermining their self-perceptions, life attitudes, personal beliefs, and value systems. As a result, in affected individuals, the sense of continuity and coherence in life may diminish or even lose meaning. Additionally, the adverse effects of cancer therapies, combined with the intense physical and psychological suffering often experienced, can lead to profound feelings of helplessness, hopelessness, and existential distress. Individuals who perceive themselves as having a life-threatening illness often report higher levels of disability, reduced social functioning, and increased mental health challenges [4]. However, for cancer patients in the terminal stages of illness, inner peace, along with spiritual and psychological well-being, may assume greater importance than physical health [5].

Acceptance of illness, along with the associated acceptance of pain, suffering, and life discomfort, represents a significant challenge for patients diagnosed with carcinoma. This is primarily due to the multifaceted impact of cancer, which affects various dimensions of a patient’s life, including physical, psychological, social, and spiritual domains [6]. Patients gradually learn to accept not only the physical symptoms of their condition but also the consequent alterations in their quality of life, including diminished self-reliance and independence. This adjustment often necessitates redefining their individual roles within their family structures and in broader society [7].

Hope, long regarded as a fundamental aspect of human life, has been demonstrated to play a significant role among cancer patients in facilitating coping mechanisms, enhancing perceived control over their illness, and promoting psychological adjustment to their condition [8,9]. Therefore, hope is a critical factor to consider in the care of cancer patients, serving as a key component in their ability to cope with adversity [10]. Although hope is widely regarded as essential to a patient’s overall well-being, it is often viewed as problematic in the context of terminal illness, particularly when it is centered on the prospect of recovery [11]. Consequently, it is essential for healthcare providers to understand the significance of hope from the perspective of patients with advanced cancer, as well as to be informed about empirically validated interventions that effectively foster hope within this population [9].

Receiving a diagnosis of lung cancer constitutes a profoundly traumatic experience for patients, affecting them biologically, psychologically, socially, and spiritually. While the existing literature includes numerous studies examining disease acceptance and the role of hope in coping among cancer patients, there remains a notable gap in the research specifically addressing both the level of illness acceptance and the experience of hope in individuals diagnosed with lung cancer. Furthermore, no studies have explored the specific hope profiles that may enhance the overall sense of hope in this patient population. Additionally, there is a lack of empirical data exploring the relationship between illness acceptance and hope, and how these variables correlate with socio-demographic and temperamental characteristics in lung cancer patients.

### Objective of This Study

The objective of this study was to ascertain the degree of acceptance of the disease among patients diagnosed with lung cancer and to characterize the profiles and dimensions of the hope experienced by said patients. The main research goal (the central research objective) was to ascertain the impact of patient acceptance of a lung cancer diagnosis on their perceived hope. The primary research problem necessitated the formulation of the following questions to facilitate its specification:

- What is the degree of acceptance of the disease by patients after a lung cancer diagnosis?
- What is the hope of the respondents, according to the NCN-36 Block test?
- What are the hope profiles of the respondents?
- How does the degree of acceptance of the disease affect hope?
- Is there a relationship, and if so, what is the relationship between the degree of acceptance of the disease by patients after a lung cancer diagnosis and socio-demographic–temperamental variables?
- Is there a relationship, and if so, what is the relationship between the hope of patients after a lung cancer diagnosis and socio-demographic–temperamental variables?

## 2. Materials and Methods

This study involved 104 patients (52.88% of whom were female and 47.12% of whom were male) who had been diagnosed with lung cancer. The majority of the study group were over 65 years old (57.69%). The vast majority of the study participants (77.88%) resided in urban areas with their families (80.77%) and were married (58.65%). The largest percentage of the respondents had received at least secondary education (41.35%) or basic vocational education (28.85%).

With regard to temperamental traits, the results were varied. The patients were asked to self-assess whether their mood was predominantly sad–unstable, sad–stable, cheerful–unstable, or cheerful–stable, and their pace or level of activity was categorized as slow–passive, slow–active, fast–passive, or fast–active. The majority of the participants exhibited a cheerful–balanced mood (39.42%) or a sad–balanced mood (32.69%). People with a cheerful–unstable mood (20.19%) and people with a sad–unstable mood (7.69%) were clear minorities. The overall pace of the respondents’ activity was also varied, with 44.23% of all respondents demonstrating a slow–active pattern, 25.96% displaying a fast–active pattern, 17.31% exhibiting a slow–passive tendency, and 12.50% manifesting a fast–passive pattern. The respondents were predominantly individuals in their retirement years, constituting 73.08% of the total sample. The majority of these individuals were under the care of their spouses (57.69%) or children (25%). Moreover, the majority of the respondents (76.87%) had adopted a new lifestyle after receiving a diagnosis of a malignant tumor, with the most common changes including modified eating habits, smoking cessation, and the adoption of regular physical activity within their capabilities.

This study used the Personal Card (KI), Block’s Hope Scale (NCN-36), and the Acceptance of Illness Scale (AIS). The Personal Card (KI) is a set of data that was selected from an extensive individual sheet developed by Witkowski [12] and was supplemented with new information tailored to the specifics of the current research. The primary goal of developing the Personal Card was to collect structured information about the patient and their immediate environment. When designing this tool, emphasis was placed on the simplicity and accessibility of the information (which are particularly important in the context of conducting surveys). In addition to basic demographic data such as age, gender, place of residence, marital status, and educational background, variables related to the emotional and motivational sphere were included. These comprised the general mood, sense of inner peace, and ease of expressing emotions.

The AIS was designed to evaluate adults who are currently experiencing health issues. The AIS includes 8 statements that reflect the negative effects of poor health, such as limitations caused by the illness, reduced independence, feelings of reliance on others, and lowered self-worth. For each statement, patients assess their current condition using a 5-point scale ranging from 1 (difficulty in adapting to the illness) to 5 (high acceptance of the illness). The total acceptance score, calculated by summing the points across all statements and ranging from 8 to 40, reflects the individual’s level of adaptation, with lower scores indicating poor adjustment and distress and higher scores signifying better acceptance and emotional well-being [13]. In studies conducted on cancer patients in Poland, the reliability of the AIS was determined to be high, with Cronbach’s alpha values of 0.871 [14] and 0.86 [6].

Block’s Hope Scale (NCN-36) is a psychological tool designed to measure hope in people facing serious, life-threatening illnesses. It contains 36 items rated on a 7-point Likert scale and includes four main subscales (each with 8 items) plus four buffer questions. This scale assesses hope across four dimensions: situational (related to treatment and trust in medicine), telic–temporal (focused on future goals and dreams), spiritual–religious (trust in God or a higher power), and affective (emotional resilience and inner peace). This research tool was also presented in previously published articles [15,16,17,18]. These dimensions reflect different aspects of hope from confidence in recovery to spiritual support and emotional stability. High scores indicate stronger hope. Scores are interpreted on a scale from 1 to 7, where higher numbers reflect stronger hope, and the exact interpretation of the results is as follows: 1.0–1.99—hopelessness; 2.0–2.99—very poor hope; 3.0–3.99—poor hope; 4.0–4.99—moderate hope; 5.0–5.99—strong hope; 6.0–7.0—very strong hope. Each individual’s profile is based on an average of the results achieved across all four subscales.

In the situational dimension, low hope may lead to a loss of faith in therapy, distrust in medical staff, and poor cooperation. Moderate hope reflects partial trust, while high hope fosters a strong belief in recovery and active engagement in treatment. Excessive hope, however, may indicate denial of the illness’s seriousness.

In the telic–temporal dimension, low hope can result in a lack of motivation to begin or continue treatment. A moderate level suggests some willingness to follow recommendations, while high hope promotes strong commitment and cooperation. Excessive hope may reflect unrealistic expectations.

In the spiritual–religious dimension, low hope may lead to a loss of life’s meaning. Moderate hope shows some sense of purpose, while strong hope supports acceptance, spiritual peace, and resilience. Excessive hope may signal apathy or passive resignation.

In the affective dimension, weak hope may leave patients overwhelmed by fear. A moderate level indicates some emotional strength, while strong hope helps reduce sadness, anxiety, and distress in the face of illness and uncertainty.

This tool demonstrates high reliability, with a Cronbach’s alpha of 0.92 for the overall score and values ranging from 0.72 to 0.86 for the individual subscales: 0.84 for the situational dimension, 0.72 for the telic–temporal dimension, 0.86 for the spiritual–religious dimension, and 0.82 for the affective subscale.

The criteria for selecting patients for this study were the patient’s consent, being diagnosed with lung cancer, and being 18 years old or older. The patients also needed to display functional capacity in the cognitive sphere and an ability to think logically. The criteria for excluding patients from this study were a lack of patient consent, being younger than 18 years of age, disorders of logical thinking, and no diagnosis of lung cancer. This study took in all patients who agreed to participate, met the required criteria, and correctly completed the questionnaires containing the research tools. It was conducted from 1 July 2024 to 31 December 2024 in the tuberculosis and lung diseases ward of the lung diseases department of the Stefan Cardinal Wyszyński Provincial Specialist Hospital in Lublin. The consent to conduct this research was obtained from the Bioethics Committee at the Medical University of Lublin (Annex to Order No. 148/2023 of the Rector of the Medical University of Lublin of 24 November 2023) (No. KE/503/06/2024).

### Statistical Analysis

Statistical analysis was performed using Statistica 13 [TIBCO Software Inc., Kraków, Poland (2017), Statistica (data analysis software system), version 13, http://tibco.com]. The group of patients was characterized via counts and percentages in terms of socio-demographic–temperamental variables. The results of the AIS and the NCN-36 scale were presented using descriptive statistics such as means, standard deviations, medians, first quartiles, third quartiles, modes, minimums, and maximums. Hierarchical cluster analysis, using agglomeration with the Ward method and the Euclidean distance as a similarity measure, was performed to distinguish groups of lung cancer patients who differed in their acceptance of their own disease and their experienced hope. Variables describing illness acceptance and hope in the situational, telic–temporal, spiritual–religious, and affective dimensions were subjected to cluster analysis after standardization to ensure the comparability of the values. The use of Ward’s method and the Euclidean distance is the standard and recommended approach in hierarchical cluster analysis when the goal is to obtain well-defined, interpretable clusters from multivariate quantitative data [19,20]. Ward’s method aims to create clusters that are maximally homogeneous internally while being distinct from one another. The Euclidean distance was adopted as the similarity measure because it is compatible with Ward’s method, which is based on minimizing the total within-cluster sum of squared deviations. Moreover, the Euclidean distance is a natural and intuitive metric in multidimensional space. When data are standardized, it ensures that all variables contribute equally to the clustering process, thus preventing any single variable from disproportionately influencing the cluster structure. The number of clusters was determined based on visual inspection of a tree diagram (particularly “large gaps” in the linkage distance) and an assessment of the interpretability and psychometric significance of the resulting clusters. In addition, the Kruskal–Wallis test was employed to compare the resulting clusters in terms of the level of disease acceptance and experienced hope. For statistically significant results, Dunn’s post hoc test with Bonferroni correction was additionally applied. Nonparametric statistics were chosen due to the distribution of the variables in the groups being significantly different from a normal distribution (*p* < 0.05 for the Shapiro–Wilk test). Moreover, the relationship between disease acceptance and hope in lung cancer patients was examined using Pearson’s correlation coefficient, with statistical significance tested against the null hypothesis that the population correlation (ρ) equals zero.

Finally, backward stepwise multiple regression was used to analyze the association between illness acceptance/perception of hope and socio-demographic–temperamental characteristics. All independent variables were binary (0–1), and their initial number was 12: place of residence, age, gender, living condition, education, financial status, social and professional status, basic mood (unstable/stable and cheerful/sad), overall pace of activity (passive/active and slow/fast), and change in habits after diagnosis. The analysis began with the full model including all 12 binary independent variables. The criterion for entering the model was an F statistic ≥ 4, and the criterion for removal was an F < 3. The use of an entry threshold of F = 4 and a removal threshold of F = 3 ensured an appropriate level of rigor in eliminating weak predictors while also preventing the premature exclusion of variables with potential predictive value. These thresholds were consistent with commonly applied criteria for variable selection in stepwise regression procedures, corresponding to *p*-values of approximately 0.05 and 0.10, respectively. This approach was supported by classical sources on regression modeling [21,22]. A total of 12 steps were performed, and the process was terminated when no further variables met the criterion for removal. The tested models were compared using the F test for model reduction, assessing the significance of the regression coefficients.

## 3. Results

The results obtained from this study concern the degree of disease acceptance by patients after a diagnosis of lung cancer and the characteristics of the hope they experience.

### 3.1. The Acceptance of the Disease by Patients After a Diagnosis of Lung Cancer and Their Hope

The conducted study, employing the AIS and the NCN-36 test, demonstrates that the acceptance of the disease in the studied group of patients was at an average level (23.14 ± 8.29). Conversely, the level of hope in three of the four dimensions (namely the situational, telic–temporal, and spiritual–religious dimensions) was elevated, while the affective aspect was average. It is important to emphasize, however, that the overall result for the hope experienced by the patients was high. Detailed descriptive statistics regarding the study results are presented in Table 1.

### 3.2. Characteristics of Cluster Analysis of Patients

It is evident from the research results that clusters of patients are distinguishable in terms of their acceptance of the disease and their experience of hope. In order to achieve the objectives of this study, a cluster analysis was thus performed via agglomeration, with the Ward method and the Euclidean distance being utilized. The number of clusters was determined based on a tree diagram (Figure S1) and a plot of the linkage distances across the clustering steps (Figure S2). In both plots, a large gap in linkage distance was found, and the tree diagram was cut just below this gap.

The analysis yielded four distinct clusters of patients. The patients from the resulting clusters differed significantly in terms of disease acceptance and hope across the situational, telic–temporal, spiritual–religious, and affective dimensions (*p* < 0.001), with a strong effect size (η^2^ > 0.14).

Cluster 1 comprised patients who exhibited relatively low levels of acceptance of their lung cancer diagnosis. These individuals demonstrated a moderate perception of hope in the telic–temporal, spiritual–religious, and situational dimensions and a low level of affective hope.

Cluster 2 included patients characterized by a moderate level of disease acceptance and the lowest levels of hope across all measured dimensions.

Cluster 3 encompassed the patients with the lowest degree of disease acceptance. Despite this, they reported high levels of hope in the situational, telic–temporal, and spiritual–religious domains and a moderate level of affective hope.

Cluster 4 consisted of the patients with the highest levels of disease acceptance and the highest levels of hope across all dimensions.

A detailed summary of the cluster analysis is provided in Table 2 below.

**Figure 1:**
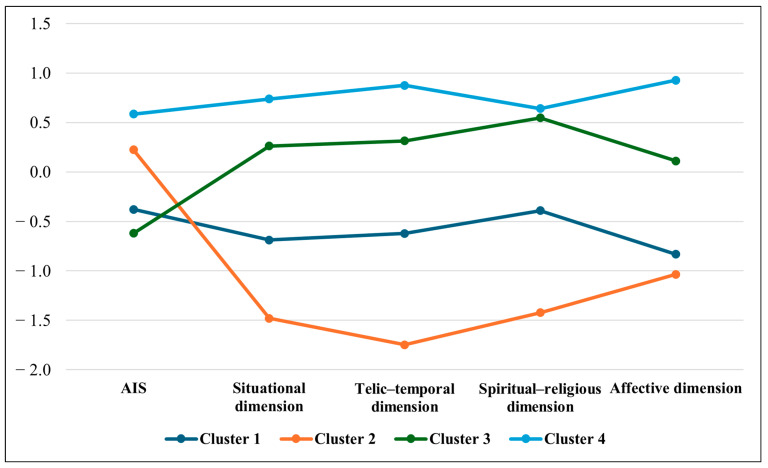
The cluster analysis of the results of the AIS and the NCN-36 test (standardized data and medians).

The outcomes of the Acceptance of Illness Scale (AIS) and the NCN-36 questionnaire are illustrated in Figure 1 so as to enhance the clarity and interpretability of the cluster analysis results.

Table 3 presents the characteristics of the patients in the individual clusters. These clustered patients differed significantly in terms of age and social and professional status (*p* < 0.0056 after Bonferroni correction). Cluster 1 comprised patients with low illness acceptance; average levels of situational, telic–temporal, and spiritual–religious hope; and low affective hope. Moreover, a high proportion were over 75 years old (31.82%) and professionally inactive (90.91%). A similar pattern was observed in Cluster 3 (low illness acceptance and high hope), where 20.0% of the patients were over 75 and all were professionally inactive. In contrast, Clusters 2 and 4 included significantly more patients aged 75 or younger who were professionally active. Cluster 1 also had the highest percentage of rural residents (40.91%) and the lowest proportion of individuals with higher education (4.55%). Cluster 3, in addition, had the highest share of patients reporting average or poor financial situations (58.82%) and the lowest percentages of individuals with an unstable mood (12.0%) and passive behavior (20.0%). Cluster 4, in contrast, stood out for having the highest proportion of patients with a stable mood (77.5%) and those exhibiting high levels of activity (fast–passive and fast–active patterns) (47.5%).

### 3.3. The Impact of the Patients’ Levels of Hope on Disease Acceptance

A weak yet statistically significant positive correlation was identified between disease acceptance and the level of hope in the affective dimension (r = 0.338; *p* < 0.001). An increase in hope in the affective dimension of 1, according to the NCN-36 questionnaire, caused an increase in illness acceptance of 1.84 on the AIS. No statistically significant correlations were observed for the remaining dimensions of hope. Detailed data are presented in Table 4.

### 3.4. The Relationships Between the Degree of Disease Acceptance in Patients Diagnosed with Lung Cancer and Socio-Demographic–Temperamental Variables

The relationships between the degree of disease acceptance among patients diagnosed with lung cancer and various socio-demographic and temperamental variables were also investigated using backward stepwise multiple regression analysis. The independent variables taken into account included place of residence, age, gender, living condition, education, financial status, social and professional status, basic mood (unstable/stable and cheerful/sad), overall pace of activity (passive/active and slow/fast), and change in habits after diagnosis. Age, place of residence, and education were found to have a significant impact on disease acceptance, with disease acceptance being lower in patients over the age of 75 (b = −6.180, *p* = 0.006) and higher in patients with higher education (b = 5.918, *p* = 0.011) and those living in urban areas (b = 3.837, *p* = 0.038). On average, the level of disease acceptance was 6.18 points lower on the AIS in patients aged over 75, 3.84 points higher in urban residents, and 5.92 points higher in patients with higher education. Detailed results are presented in Table 5.

### 3.5. The Relationships Between the Different Dimensions of the Perception of Hope in Lung Cancer Patients and Their Socio-Demographic–Temperamental Characteristics

Table 6, Table 7, Table 8, Table 9 and Table 10 present the relationships between the perception of hope across various dimensions and the socio-demographic and temperamental characteristics of patients diagnosed with lung cancer. Backward stepwise multiple regression analysis was applied, taking into account independent variables such as place of residence, age, gender, living condition, education, financial status, social and professional status, basic mood, overall pace of activity, and change in habits after diagnosis. Basic mood was found to significantly influence all dimensions of hope, with higher levels of hope observed in patients with a stable mood. Additionally, individuals who were very active demonstrated a stronger sense of hope in the situational and affective dimensions, as well as overall. Professional activity positively correlated with situational hope, while patients living alone tended to exhibit greater spiritual–religious hope. Furthermore, being under 75 years of age, residing in an urban area, and having a cheerful mood were all associated with higher levels of affective hope.

## 4. Discussion

Lung cancer is the most common malignant tumor in the world and one of the cancers with the most severe prognosis. Acceptance of illness, especially cancer, is the most important element of the adaptation process: the better the acceptance of cancer, the lower the stress level and the higher the patient’s self-esteem [23]. Acceptance of cancer has long been regarded as a key factor in patients’ psychological adjustment to the illness. Research to date indicates that cancer acceptance plays a crucial role in interventions designed to reduce both general and cancer-specific suffering in oncology patients. The researchers emphasize that studies on patients’ attitudes toward cancer should incorporate the development of multifaceted measures of acceptance and identification that are grounded in theories of psychological and social processes that foster greater disease acceptance [24].

A lack or a low level of acceptance has been associated with increased symptom severity and negatively impacts the patient’s biological, psychological, social, and spiritual well-being. Research has demonstrated a positive correlation between disease acceptance and life satisfaction among cancer patients.

The degree to which a patient accepts the disease is influenced by factors such as the specific type of cancer and recent exposure to chemotherapy within the preceding months [6]. Acceptance of the diagnosis plays a vital role in enhancing psychological well-being. Empirical studies have identified positive correlations between a sense of meaning in life and acceptance of cancer, with reported correlation coefficients ranging from r = 0.19 to r = 0.38 [25]. Previous research has demonstrated a significant correlation between the level of cancer acceptance and the severity of symptoms such as pain, fatigue, and diarrhea [26].

Studies conducted at the Maria Sklodowska-Curie Institute—Oncology Center in Warsaw, involving a sample of 1187 patients diagnosed with malignant cancer, utilized the Acceptance of Illness questionnaire developed by B. J. Felton, T. A. Revensson, and G. A. Hinrichsen. The findings indicate that both socio-economic factors (such as education, place of residence, income, and employment status) and medical factors (including the presence of metastases and the type of treatment administered) significantly influence patients’ levels of illness acceptance [13]. Numerous studies indicate that the level of disease acceptance among patients varies, with reported scores ranging from 21.6 ± 7.32 to 30.39 ± 8.11 [6,23,26,27,28].

Hope plays a significant role in helping patients cope with the negative emotions induced by cancer. However, a review of the scientific literature reveals that only two articles to date have specifically addressed the importance and value of hope in lung cancer patients’ adaptation to life with the disease. Researchers from Duke University Medical Center in Durham, NC, USA, found that hope plays a crucial role in explaining the variability in how patients adjust to lung cancer. In their cross-sectional study, the authors examined how hope, as conceptualized by Snyder et al., interacts with various indices of adjustment to lung cancer. The data analysis from this study reveals that hope is inversely associated with key symptoms of lung cancer (such as pain, fatigue, and cough) and psychological distress (including depression). Thus, hope plays a crucial role in patients adjusting to lung cancer. Although the study sample was relatively small (n = 51), it is important to note that the findings are relevant to the development of comprehensive care plans for these patients [29].

Original research on the value of hope for survival in advanced non-small-cell lung cancer, focusing on patient and physician preferences, was conducted by a team of researchers from RTI Health Solutions (Research Triangle Park, NC, USA) and Bristol Myers Squibb (Princeton, NJ, USA) using specially developed survey instruments for patients and physicians. The study revealed a potentially significant gap between physicians’ and patients’ perspectives regarding survival. The physicians placed greater importance on increases in expected survival than the patients. This research underscores the importance of considering patient priorities and engaging in shared decision-making when selecting treatment options [30].

The meaning and value of hope in cancer patients have been explored by several research teams. In relation to our study, the findings presented in previously published scientific articles on this topic are particularly relevant.

The studies aimed to examine the relationships between various attitudes and the level of hope in cancer patients [31]; the influence of the disease status on the level of hope in cancer patients experiencing pain, differences in the level of hope between cancer patients with and without pain, and which dimensions of pain are associated with hope [32]; the level of hope in cancer patients, differences in hope levels during and after hospitalization, and the correlations between hope, quality of life, and selected disease symptoms [8]; the level of hope of newly diagnosed oncology patients and the correlations between the level of hope and gender, age, educational attainment, and type of cancer [9]; the relationship between hope and life and death in oncological patients with a prognosis of death within a few weeks [33]; the associations between quality of life in cancer patients and hope, a sense of meaning in life, and spiritual struggles (particularly in the religious domain) [34]; and the influence of hope and social support on patients’ resilience in coping with their illness [35]. Additionally, the purpose of these studies was to explore whether there is a difference in the level of hope between oncological patients receiving curative and palliative treatments, to identify important sources of hope for these patients, and to indicate how their hope evolves over time [10], as well as to indicate to what extent hope can be a vital resource in the lives of individuals with cancer and can enhance their ability to cope during periods of suffering and uncertainty [36].

The above-mentioned studies presented the following findings: Women exhibit lower levels of hope compared to men, while hope levels are positively correlated with both a younger age and higher educational attainment [31]. The cognitive dimension of pain (i.e., the meaning attributed to pain) of oncological patients is significantly correlated with hope, whereas the sensory dimensions (such as pain intensity and relief) do not show a similar correlation [32]. Patients exhibit moderate levels of hope, with no significant difference in the hope levels between the hospital and home settings. Their hope has positive correlations with quality of life, self-esteem, coping mechanisms, adjustment to the disease, overall well-being, and comfort during hospitalization, as well as satisfaction with information provided and the support given by family, healthcare workers, and friends [8]. Older oncological patients tend to exhibit greater hope for a cure [36]. Hope is a vital resource in the lives of individuals with cancer that supports their ability to cope during periods of suffering and uncertainty [9]. Lower levels of hope exist among younger cancer patients and those living alone, and age, educational attainment, and cancer type are associated with specific dimensions of hope in newly diagnosed cancer patients [33]. Quality of life in cancer patients is positively associated with both hope and a sense of meaning in life and negatively associated with spiritual struggles, particularly in the religious domain [34]. Both hope and social support exert positive and statistically significant effects on resilience among cancer patients [35].

Review articles were also published on the basis of the CINAHL Plus^®^, MEDLINE, and PsycINFO online databases to inform meta-analyses of hope in cancer patients. The analyses revealed that while nursing intervention programs have demonstrated positive effects on hope in adults newly diagnosed with cancer, in those experiencing a first recurrence, individuals with terminal illness, and childhood cancer survivors, the current body of research remains limited, necessitating further investigation. Four key areas for future research have been identified: assessing the level of hope in cancer patients, understanding how individuals cope with a cancer diagnosis, identifying commonly used strategies to maintain hope, and evaluating nursing interventions that support the maintenance and enhancement of hope [37,38]. Also noteworthy is a meta-analysis of multiple scientific studies examining the perception of hope among patients with terminal illness. This study also highlighted some essential attributes of the concept of hope. These include positive expectation, personal qualities, spirituality, goals, comfort, help and caring, interpersonal relationships, a sense of control, legacy, and life review [39].

The literature review confirmed the researchers’ hypothesis that no previous studies had simultaneously examined both the level of disease acceptance and the experience of hope in patients following a diagnosis of lung cancer. As outlined above, existing publications tend to address each of these variables in isolation. The present study aimed to explore the relationships between these two psychological constructs and a range of socio-demographic and temperamental variables. This objective was considered particularly significant, as identifying such relationships may offer valuable insights for the development and implementation of comprehensive, interdisciplinary care strategies for this patient population. A diagnosis of cancer invariably constitutes a profound psychological shock, often leading to disorganization across multiple dimensions of life: biological, psychological, social, and spiritual dimensions. Investigating how these disruptions manifest in the lived experiences of patients can inform practical, immediate interventions to support individuals who frequently feel overwhelmed, fearful, and isolated in the wake of their diagnosis.

While many existing studies primarily focus on reporting statistical outcomes, there remains a lack of practical guidelines derived from such findings—guidelines that would assist healthcare professionals in providing targeted, meaningful support. The authors of the present study therefore sought not only to assess the level of illness acceptance among lung cancer patients but also to examine the nature and extent of the hope they experience—a psychological resource widely recognized as essential to human resilience and coping. By identifying areas in which hope is diminished, this study provides a foundation for the development of tailored interventions aimed at strengthening this resource. The standardized research instruments employed in this study enabled a deeper discussion of actionable strategies for psychosocial support, ultimately contributing to a more holistic approach to patient care.

Each examined area and indicator provided information on what oncological patients need or expect. As their expectations were sometimes unrealistic, they also indicated what kind of help and care is genuinely required. The individual items and dimensions direct researchers very precisely to such actions.

The results of the conducted study revealed that the level of disease acceptance among the patients diagnosed with lung cancer was moderate, with a mean score of 23.14 (SD = 8.29). In contrast, the level of hope experienced by these patients was generally high in the situational, telic–temporal, and spiritual–religious dimensions, while it remained moderate in the affective dimension. Notably, the overall level of hope reported by the respondents was high. These findings suggest that despite a moderate level of acceptance of their illness, the patients maintained a strong sense of hope regarding their recovery or an improvement in their health, the effectiveness of their treatment, and their confidence in the competence of medical professionals, as well as in the efficacy of the modern medical elements reflected in the situational dimension of hope. Additionally, the respondents reported a high level of telic–temporal hope, indicating that they found meaning in life; believed that many valuable experiences still awaited them; and remained focused on achieving important personal goals, fulfilling plans, and realizing dreams. This dimension of hope thus appeared to serve a motivational function, encouraging the patients to engage actively in efforts to restore their health.

The high level of spiritual–religious hope observed among the participants further indicated a reliance on religious faith and a belief in a transcendent reality beyond death. The patients expressed trust in God and drew strength from spiritual convictions, which provided existential comfort and a broader perspective on their illness experience. However, the moderate level of hope in the affective dimension suggests that the patients were not entirely free from emotional distress. Many experienced episodes of fear, uncertainty, sadness, depression, and inner anxiety, highlighting the emotional vulnerability that often accompanies a life-threatening diagnosis. These findings underscore the importance of addressing both the cognitive and emotional dimensions of hope in psychosocial interventions for individuals coping with a serious illness.

To provide a more comprehensive understanding of the levels of disease acceptance and hope experienced by the respondents, the authors identified four distinct patient profiles based on the data obtained. The first profile, referred to as the ‘non-accepting–fearful group’, comprised individuals who demonstrated a low level of acceptance of their condition following a lung cancer diagnosis yet maintained a relatively optimistic outlook toward the future, believing they could still pursue personal goals and aspirations. Despite their future-oriented expectations, their low level of acceptance undermined the motivational role of hope, limiting their engagement in proactive efforts to improve their health. The patients in this group exhibited partial trust in God. Hence, they did not fully ground their hope in religious faith. Similarly, they expressed ambivalence regarding the potential for improvement in their health, the effectiveness of the prescribed therapy, and confidence in modern medical treatments. Emotionally, these individuals were marked by vulnerability, frequently experiencing fear, persistent uncertainty, sadness, depressive symptoms, and internal anxiety. This profile suggests a discordance between cognitive hope for the future and the emotional and spiritual resources needed to cope effectively with their illness.

The second group of patients was characterized by an attitude the researchers termed ‘indifferent–desperate’. The individuals in this group exhibited a profound absence of hope regarding recovery or any improvement in their health condition. They expressed a lack of belief in the effectiveness of the treatment they were receiving, as well as in the potential of modern medicine. Furthermore, they held no hope for the future and demonstrated a marked loss of motivation to engage in any efforts to combat the illness or sustain life. This group also reported a significant decline in religious or spiritual trust, having lost confidence in God. Emotionally, these patients were overwhelmed by persistent existential fear and a pervasive sense of hopelessness concerning their situation.

The third group of patients exhibited an attitude identified by the researchers as ‘non-accepting–fighting’. This group was characterized by the lowest level of disease acceptance among all participants. Moreover, they demonstrated a strong and unwavering expectation of recovery or improvement in their health status. These patients expressed full confidence in the effectiveness of the prescribed therapy and in the competence of the medical professionals overseeing their care. They maintained a clear belief that they would still be able to pursue and achieve their personal goals and aspirations, and they exhibited a high level of motivation to engage in efforts aimed at restoring their health. Furthermore, they demonstrated a deep sense of spiritual trust, placing their hope entirely in God. However, despite these optimistic convictions, these patients were not immune to emotional distress. They occasionally experienced fear and uncertainty regarding the future, which triggered internal anxiety and a sense of instability. During such moments, they struggled to maintain their psychological balance and expressed a desire to withdraw or escape from their circumstances, indicating underlying emotional vulnerability despite their outward hopefulness.

The fourth group of patients demonstrated an attitude identified by the researchers as ‘accepting–trusting’. The individuals in this group exhibited full acceptance of their illness despite the challenges associated with a cancer diagnosis. They maintained strong expectations regarding the positive outcomes of their treatment, expressing a high level of trust in the competence of their physicians and confidence in the appropriateness of the chosen therapeutic interventions. These patients held a future-oriented outlook, believing that many positive experiences still awaited them. They reported having numerous plans and aspirations, and they were highly motivated to engage in efforts aimed at restoring their health. Importantly, their hope was not limited to medical treatment; they also placed significant trust and hope in God, indicating a strong spiritual foundation. Their approach to illness was marked by emotional resilience. They did not succumb to fear, and their overall attitude toward the disease was characterized by courage, determination, and psychological strength. This profile represents a well-integrated coping strategy that combines medical trust, personal motivation, and spiritual conviction.

This study demonstrated a weak but statistically significant positive correlation between the degree of disease acceptance and the level of hope in the affective dimension (r = 0.338; *p* < 0.001). This indicates that as the patients’ acceptance of their condition increased, they were less likely to experience or be overwhelmed by fear. Consequently, their overall approach to the illness was marked by greater emotional resilience, reflected in an attitude characterized by courage and psychological strength.

The researchers also found statistically significant effects of place of residence, age, and education on disease acceptance. Multiple regression analysis indicated that urban patients showed a higher level of disease acceptance than rural residents (b = 3.837, *p* = 0.038). These disparities may be attributed to having greater access to healthcare services, including quicker access to treatment and greater availability of professional psychosocial support, which may facilitate the acceptance process. Furthermore, age was shown to significantly influence disease acceptance, with a lower level of acceptance observed among patients over 75 years of age (b = −6.180; *p* = 0.006). This suggests that older patients may face greater challenges in coming to terms with their diagnoses, potentially due to increased physical vulnerability, reduced social support, or a more pessimistic outlook on recovery and treatment outcomes. Higher education was also found to be significantly associated with greater acceptance of the disease (b = 5.919, *p* = 0.011).

In the course of the undertaken research, a multiple regression analysis with hope as the dependent variable and socio-demographic–temperamental characteristics as independent variables revealed statistically significant results. Specifically, it highlighted the positive effect of stable mood and the form or degree of activity on the perception of hope in lung cancer patients. Additionally, a higher level of situational hope was observed among professionally active patients, and higher levels of spiritual–religious hope and the general perception of hope were indicated among respondents living alone. Ages up to 75 years, living in a city, and a cheerful mood were also noted to have positive effects on the affective dimension of hope. These findings suggest that both emotional and temperamental factors, as well as socio-demographic characteristics, play important roles in shaping the experience of hope among patients.

## 5. Conclusions

Among patients diagnosed with lung cancer, the level of acceptance of their health status was found to be moderate, while the overall level of hope experienced was high. Specifically, the respondents reported elevated hope in the situational, telic–temporal, and spiritual–religious dimensions, with only moderate levels observed in the affective domain. Based on the data, four distinct psychological profiles were identified, reflecting varying degrees of disease acceptance and hope. These were non-accepting–fearful, indifferent–desperate, non-accepting–fighting, and accepting–trusting. It was also shown that increased hope in the affective dimension led to increased acceptance of the disease.

The statistically significant influences of place of residence, age, and education on disease acceptance were made evident. Additionally, individuals living alone exhibited significantly higher levels of spiritual–religious hope and general hope, while professionally active individuals had higher situational hope. Interestingly, higher levels of affective hope were observed in patients up to 75 years of age, urban residents, and those with a cheerful mood. Indeed, across all dimensions of hope, individuals with a stable mood demonstrated higher levels of hope, whereas those with an unstable mood reported lower levels. Moreover, the overall pace of undertaken activity significantly influenced the levels of hope in the situational and affective dimensions, as well as the general sense of hope.

As lung cancer patients demonstrate varying levels of acceptance of their disease and experienced hope, it is important to adapt healthcare to their psychological states. As previous research in this area has shown, both disease acceptance and the level of hope influence patients’ quality of life, the course of the disease, and their overall well-being.

### 5.1. Practical Recommendations

1. Healthcare providers should be trained to understand hope as it is perceived by patients with advanced cancer. Moreover, they should be informed about empirically validated interventions that have been shown to enhance hope within this population.
2. Nursing intervention programs should demonstrate the positive effects of fostering hope in oncology patients.
3. The focus should be on delivering information about the patients’ health conditions in a way that preserves and supports their hope, without offering unrealistic, false, or unwarranted expectations.

### 5.2. The Limitations of This Study

Further research is needed on the psychological well-being of patients with lung cancer, including at other oncology centers. The presented study did not take into account the time since receiving a lung cancer diagnosis, the cancer stage, or the methods of treatment used, which may also have significant impacts on the experience of hope and acceptance of one’s disease among oncology patients.

## Figures and Tables

**Table 1:** Descriptive statistics related to acceptance of one’s own illness and perception of hope.

Scale		Descriptive Statistics (N = 104)								
		M	SD	Me	Q1	Q3	Min	Max	Mo	Mo (n)
AIS	Overall score	23.14	8.29	23.00	18.00	29.00	8.00	40.00	20.00	12
NCN-36	Situational dimension	5.08	1.58	5.50	3.88	6.38	1.00	7.00	7.00	14
	Telic–temporal dimension	5.58	1.33	5.94	4.69	6.81	2.00	7.00	7.00	22
	Spiritual–religious dimension	5.48	1.40	5.88	4.63	6.56	1.00	7.00	6.25	16
	Affective dimension	4.46	1.52	4.63	3.25	5.69	1.00	7.00	4.75	8
	Overall score	5.15	1.25	5.45	4.33	6.25	2.00	7.00	Multiple	5

Legend: M—mean, SD—standard deviation, Me—median, Q1—first quartile, Q3—third quartile, Min—minimum, Max—maximum, Mo—mode, Mo (n)—modal frequency.

**Table 2:** Cluster analysis for the AIS and the NCN-36 scale.

Cluster Analysis	AIS	Dimensions of Hope, According to NCN-36 Scale			
	Overall Score	Situational	Telic–Temporal	Spiritual–Religious	Affective
Cluster 1 (N = 22)	20.0 (18.0–21.0) ^ab^	4.0 (3.75–5.13) ^a^	4.75 (4.38–5.38) ^a^	4.94 (4.0–6.25) ^a^	3.19 (2.50–3.63) ^a^
Cluster 2 (N = 17)	25.0 (18.0–32.0) ^ac^	2.75 (1.75–3.13) ^a^	3.25 (2.88–4.13) ^a^	3.50 (3.13–4.0) ^a^	2.88 (1.75–4.13) ^a^
Cluster 3 (N = 25)	18.0 (12.0–20.0) ^b^	5.50 (4.63–6.63) ^b^	6.0 (5.50–6.88) ^b^	6.25 (5.50–6.88) ^b^	4.63 (4.0–4.75) ^b^
Cluster 4 (N = 40)	28.0 (24.50–32.50) ^c^	6.25 (5.94–6.69) ^b^	6.75 (6.25–7.0) ^b^	6.38 (6.13–6.88) ^b^	5.88 (5.13–6.63) ^c^
Kruskal–Wallis test	H = 40.099, *p* < 0.001, η^2^ = 0.371	H = 59.234, *p* < 0.001, η^2^ = 0.562	H = 62.855, *p* < 0.001, η^2^ = 0.599	H = 50.088, *p* < 0.001, η^2^ = 0.471	H = 66.530, *p* < 0.001, η^2^ = 0.635

Legend: The median (first quartile–third quartile) is given for each group. Dunn’s post hoc test with Bonferroni correction was used, and different letters indicate statistically significant differences. The significance level was adjusted using the Bonferroni correction: α = 0.05/5 = 0.01.

**Table 3:** The characteristics of the patients in each cluster from the cluster analysis of the AIS and the NCN-36 scale.

Variable		Characteristics of Patients			
		Cluster 1	Cluster 2	Cluster 3	Cluster 4
Gender	female	10 (45.45%)	9 (52.94%)	10 (40.0%)	20 (50.0%)
	male	12 (54.55%)	8 (47.06%)	15 (60.0%)	20 (50.0%)
Place of residence	rural	9 (40.91%)	2 (11.76%)	4 (16.0%)	8 (20.0%)
	urban	13 (59.09%)	15 (88.24%)	21 (84.0%)	32 (80.0%)
Age *	up to 75 years	15 (68.18%)	16 (94.12%)	20 (80.0%)	39 (97.50%)
	over 75 years	7 (31.82%)	1 (5.88%)	5 (20.0%)	1 (2.50%)
Living	alone	5 (22.73%)	2 (11.76%)	3 (12.0%)	10 (25.0%)
	with family	17 (77.27%)	15 (88.24%)	22 (88.0%)	30 (75.0%)
Education	primary, vocational, secondary	21 (95.45%)	14 (82.35%)	22 (88.0%)	34 (85.0%)
	higher	1 (4.55%)	3 (17.65%)	3 (12.0%)	6 (15.0%)
Financial situation	very good, good	14 (63.64%)	27 (67.50%)	7 (41.18%)	17 (68.0%)
	average, poor	8 (36.36%)	13 (32.50%)	10 (58.82%)	8 (32.0%)
Social and professional status *	professionally inactive	20 (90.91%)	11 (64.71%)	25 (100.0%)	29 (72.50%)
	professionally active	2 (9.09%)	6 (35.29%)	0 (0.0%)	11 (27.50%)
Basic mood	sad–unstable	2 (9.09%)	3 (17.65%)	1 (4.0%)	2 (5.0%)
	sad–stable	8 (36.36%)	4 (23.53%)	12 (48.0%)	10 (25.0%)
	cheerful–unstable	7 (31.82%)	5 (29.41%)	2 (8.0%)	7 (17.50%)
	cheerful–stable	5 (22.73%)	5 (29.41%)	10 (40.0%)	21 (52.50%)
Overall pace of activity	slow–passive	6 (27.27%)	5 (29.41%)	4 (16.0%)	3 (7.50%)
	slow–active	8 (36.36%)	7 (41.18%)	13 (52.0%)	18 (45.0%)
	fast–passive	3 (13.64%)	2 (11.76%)	1 (4.0%)	7 (17.50%)
	fast–active	5 (22.73%)	3 (17.65%)	7 (28.0%)	12 (30.0%)

* indicates significant differences according to Fisher’s exact test; the significance level was adjusted using the Bonferroni correction: α = 0.05/9 = 0.0056.

**Table 4:** The relationship between the experience of hope, as measured by the NCN-36 questionnaire, and the degree of disease acceptance, as assessed by the AIS.

The Experience of Hope		AIS Overall Score			
		b	r	t	*p*
NCN-36	Situational dimension	0.365	0.069	0.702	0.484
	Telic–temporal dimension	0.285	0.046	0.465	0.643
	Spiritual–religious dimension	0.358	0.060	0.611	0.543
	Affective dimension	1.839	0.338	3.631	<0.001
	Overall score	1.016	0.154	1.571	0.119

**Table 5:** The relationships between the respondents’ acceptance of their condition after the diagnosis of lung cancer and their socio-demographic–temperamental characteristics.

N = 104	Regression Summary for Dependent Variable: Acceptance of Lung Cancer Disease R = 0.40175322, R^2^ = 0.16140565, Adjusted R^2^ = 0.13624782, F(3100) = 6.4157, *p* < 0.00051, Std. Error of Estimate: 7.7015					
	b*	Std. Err. of b*	b	Std. Err. of b	t(100)	*p*-Value
Intercept			20.24798	1.642961	12.32408	0.000000
Age (0—up to 75, 1—over 75)	−0.255757	0.091597	−6.17955	2.213139	−2.79221	0.006273
Place of residence (0—rural, 1—urban)	0.193100	0.091749	3.83698	1.823083	2.10467	0.037827
Education (0—primary/vocational/secondary, 1—higher)	0.237314	0.091767	5.91758	2.288283	2.58604	0.011149

b*: standardized regression coefficient; b: unstandardized regression coefficient.

**Table 6:** The relationships between the situational hope experienced by the respondents after the diagnosis of lung cancer and their socio-demographic–temperamental characteristics.

N = 104	Regression Summary for Dependent Variable: Situational Dimension of Hope R = 0.41902931, R^2^ = 0.17558556, Adjusted R^2^ = 0.14227589 F(4,99) = 5.2713, *p* < 0.00068, Std. Error of Estimate: 1.4603					
	b*	Std. Err. of b*	b	Std. Err. of b	t(100)	*p*-Value
Intercept			3.994883	0.518831	7.69978	0.000000
Living condition (0—alone, 1—with family)	−0.189301	0.096202	−0.753708	0.383031	−1.96775	0.051895
Basic mood (0—unstable, 1—stable)	0.254400	0.094564	0.890212	0.330905	2.69023	0.008382
Overall pace of activity (0—slow, 1—fast)	0.224101	0.094636	0.722818	0.305240	2.36803	0.019826
Social and professional status (0—professionally active, 1—professionally inactive)	0.234056	0.093898	0.950471	0.381310	2.49265	0.014339

b*: standardized regression coefficient; b: unstandardized regression coefficient.

**Table 7:** The relationships between the telic–temporal hope experienced by the respondents after the diagnosis of lung cancer and their socio-demographic–temperamental characteristics.

N = 104	Regression Summary for Dependent Variable: Telic–Temporal Dimension of Hope R = 0.36551288, R^2^ = 0.13359967, Adjusted R^2^ = 0.11644322 F(2101) = 7.7871, *p* < 0.00072, Std. Error of Estimate: 1.2538					
	b*	Std. Err. of b*	b	Std. Err. of b	t(100)	*p*-Value
Intercept			4.646635	0.267949	17.34146	0.000000
Financial situation (0—average/bad, 1—good/very good)	0.167101	0.093519	0.458173	0.256419	1.78681	0.076968
Basic mood (0—unstable, 1—stable)	0.302774	0.093519	0.896250	0.276828	3.23757	0.001631

b*: standardized regression coefficient; b: unstandardized regression coefficient.

**Table 8:** The relationships between the spiritual–religious hope experienced by the respondents after the diagnosis of lung cancer and their socio-demographic–temperamental characteristics.

N = 104	Regression Summary for Dependent Variable: Spiritual–Religious Dimension of Hope R = 0.37491827, R^2^ = 0.14056371, Adjusted R^2^ = 0.11478062 F(3100) = 5.4518, *p* < 0.00164, Std. Error of Estimate: 1.3127					
	b*	Std. Err. of b*	b	Std. Err. of b	t(100)	*p*-Value
Intercept			5.499822	0.331024	16.61458	0.000000
Living condition (0—alone, 1—with family)	−0.282882	0.096725	−0.996613	0.340769	−2.92460	0.004267
Basic mood (0—unstable, 1—stable)	0.265498	0.095984	0.822066	0.297198	2.76605	0.006759
Overall pace of activity (0—slow, 1—fast)	0.178075	0.094803	0.508227	0.270569	1.87837	0.063244

b*: standardized regression coefficient; b: unstandardized regression coefficient.

**Table 9:** The relationships between the affective hope experienced by the respondents after the diagnosis of lung cancer and their socio-demographic–temperamental characteristics.

N = 104	Regression Summary for Dependent Variable: Affective Dimension of Hope R = 0.45587987, R^2^ = 0.20782646, Adjusted R^2^ = 0.15882603 F(6,97) = 4.2413, *p* < 0.00078, Std. Error of Estimate: 1.3979					
	b*	Std. Err. of b*	b	Std. Err. of b	t(100)	*p*-Value
Intercept			2.257319	0.568479	3.97081	0.000138
Age (0—up to 75, 1—over 75)	−0.207604	0.094318	−0.922594	0.419151	−2.20110	0.030101
Place of residence (0—rural, 1—urban)	0.189998	0.091417	0.694389	0.334103	2.07837	0.040316
Basic mood (0—unstable, 1—stable)	0.230138	0.094237	0.778428	0.318750	2.44213	0.016412
Basic mood (0—cheerful, 1—sad)	0.225956	0.099281	0.698495	0.306905	2.27593	0.025049
Overall pace of activity (0—slow, 1—fast)	0.224486	0.100199	0.699890	0.312393	2.24041	0.027346
Social and professional status (0—professionally active, 1—professionally inactive)	0.167391	0.095091	0.657062	0.373261	1.76033	0.081504

b*: standardized regression coefficient; b: unstandardized regression coefficient.

**Table 10:** The relationships between the overall hope experienced by the respondents after the diagnosis of lung cancer and their socio-demographic–temperamental characteristics.

N = 104	Regression Summary for Dependent Variable: Overall Hope R = 0.40357536, R^2^ = 0.16287307, Adjusted R^2^ = 0.13775926 F(3100) = 6.4854, *p* < 0.00047, Std. Error of Estimate: 1.1638					
	b*	Std. Err. of b*	b	Std. Err. of b	t(100)	*p*-Value
Intercept			4.914446	0.293469	16.74605	0.000000
Living condition (0—alone, 1—with family)	−0.233657	0.095461	−0.739460	0.302108	−2.44766	0.016121
Basic mood (0—unstable, 1—stable)	0.307021	0.094730	0.853943	0.263481	3.24101	0.001618
Overall pace of activity (0—slow, 1—fast)	0.220910	0.093564	0.566351	0.239873	2.36105	0.020162

b*: standardized regression coefficient; b: unstandardized regression coefficient.

## Data Availability

The raw data supporting the conclusions of this article will be made available by the authors on request.

## Supplementary Materials

The following supporting information can be downloaded at https://www.mdpi.com/article/10.3390/jcm14124356/s1, Figure S1: Cluster analysis—tree diagram for 104 cases; Figure S2: Cluster analysis—plot of linkage distances across steps.

## Abbreviations

The following abbreviations are used in this manuscript:

AIS	Acceptance of Illness Scale
KI	Personal Card
NCN-36	Block’s Hope Scale
