# Supplementary figures and images for: The Degree of Acceptance of the Disease by Patients After a Diagnosis of Lung Cancer and Their Hope
Source: J Clin Med. 2025 Jun 18;14(12):4356. doi: 10.3390/jcm14124356 (PMC12194530; doi:10.3390/jcm14124356)

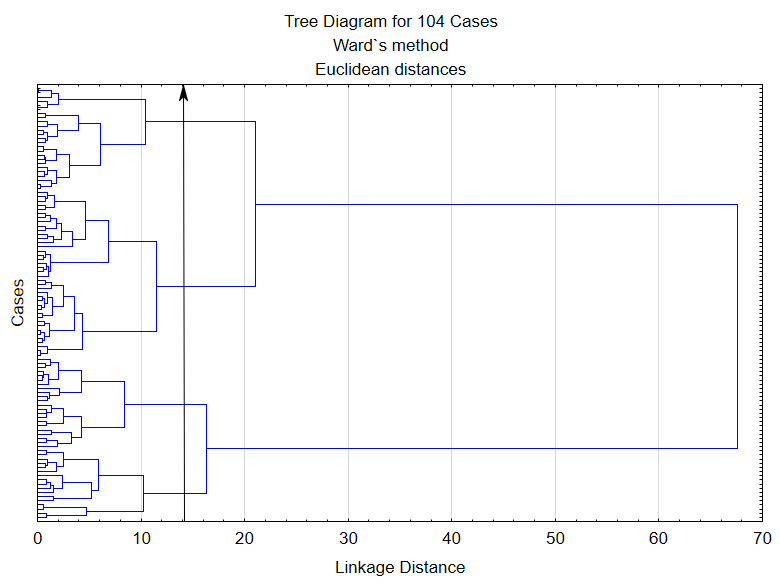

Supplement: Supplementary file 1 [file jcm-14-04356-s001.zip › Figure S1. Cluster analysis - tree diagram for 104 cases.png]

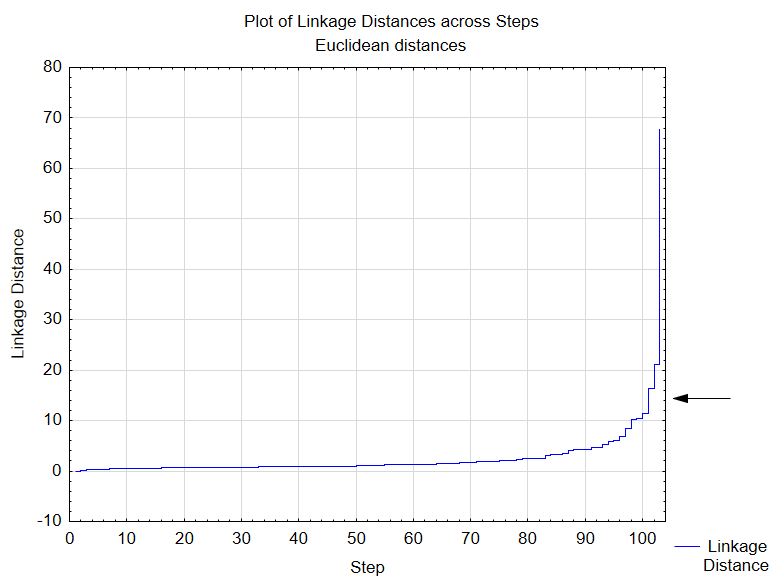

Supplement: Supplementary file 1 [file jcm-14-04356-s001.zip › Figure S2. Cluster analysis - plot of linkage distances across steps.png]
